# Generation of High Quality Memory B Cells

**DOI:** 10.3389/fimmu.2021.825813

**Published:** 2022-01-12

**Authors:** Takeshi Inoue, Ryo Shinnakasu, Tomohiro Kurosaki

**Affiliations:** ^1^Laboratory of Lymphocyte Differentiation, WPI Immunology Frontier Research Center, Osaka University, Osaka, Japan; ^2^Center for Infectious Diseases Education and Research, Osaka University, Osaka, Japan; ^3^Laboratory for Lymphocyte Differentiation, RIKEN Center for Integrative Medical Sciences, Kanagawa, Japan

**Keywords:** memory B cell, germinal center, vaccine, broadly neutralizing antibody, BCR affinity

## Abstract

Protection against pathogen re-infection is mediated, in large part, by two humoral cellular compartments, namely, long-lived plasma cells and memory B cells. Recent data have reinforced the importance of memory B cells, particularly in response to re-infection of different viral subtypes or in response with viral escape mutants. In regard to memory B cell generation, considerable advancements have been made in recent years in elucidating its basic mechanism, which seems to well explain why the memory B cells pool can deal with variant viruses. Despite such progress, efforts to develop vaccines that induce broadly protective memory B cells to fight against rapidly mutating pathogens such as influenza virus and HIV have not yet been successful. Here, we discuss recent advances regarding the key signals and factors regulating germinal center-derived memory B cell development and activation and highlight the challenges for successful vaccine development.

## Introduction

Humoral immunological memory, the basis of antibody (Ab)-based vaccination, is critical for protection against pathogen re-infection, which is largely mediated by two cellular compartments, long-lived plasma cells and memory B cells. Early memory B cells emerge after the initial immunization are primarily composed of IgM-expressing B cells harboring a small number of somatic hypermutation (SHM), whereas subsequent memory B cell development occurs in the germinal center (GC), the primary site in which iterative rounds of SHM and subsequent selection of affinity-matured B cell clones take place (1–3).

Since long-lived plasma cells are producing highly-selected and high affinity Abs for the primary antigen, such pre-existing Abs act as a first line of defense against reinfection by homologous pathogens. On the other hand, selection for memory B cells is less stringent, therefore, it has been predicted that memory B cells rather participate in defense against challenge by related pathogens or variant pathogens that escape the long-lived plasma cell-mediated defense. Indeed, memory B cells have been found to be differentiated from lower affinity precursor GC B cells, in contrast to long lived plasma cells, which arise from highly selected and high affinity cells (4–7). This probably allows memory B cells to maintain flexibility in their responsiveness to variant and related antigens. Recently, the above prediction has been directly proven. First, studies using mouse infection models (West Nile and influenza viruses) have provided clear evidence for involvement of memory B cells in heterosubtypic immunity, i.e., cross-protection to a different viral serotype than the ones in the primary infection (8, 9). Second, in the case of pandemic 2009 H1N1 influenza vaccination, individuals who had low levels of pre-existing Abs to this novel vaccine could generate broadly reactive Abs from memory B cells (10).

Given such importance of memory B cells, a vaccination strategy involving iterative exposure to cross-reactive viral antigens has been designed to elicit broadly reactive memory B cells capable of mediating heterosubtypic immunity against mutating pathogens. However, in the case of influenza vaccination, its potential efficacy seems to be limited by the inefficiency with which memory B cells enter the GC. For instance, in the case of homotypic re-challenge, the secondary GC response is largely derived from naïve, and not from memory B cells. This is likely one of the major reasons why vaccines against influenza viruses have not yet been highly successful. In this review, we first discuss recent advances in our understanding of how GC responses take place and generate memory B cells. Furthermore, mainly emphasizing the influenza system, we discuss how broadly protective memory B cells are generated, here particularly focusing on anti-stem Abs, why they cannot be efficiently induced by the current vaccination method, and the potential way to overcome these obstacles. Although we briefly touch upon pre-GC processes, more thorough reviews of these topics are available elsewhere (11, 12). Likewise, differentiation of GC B cells to plasma cells has been extensively reviewed in recent years (3, 13) and is beyond the scope of this article.

## GC Responses

A major challenge in understanding humoral immunity is to decipher how affinity maturation takes place during responses to infections or vaccine antigens. While the emphasis on GC studies has long been to understand how they support maturation towards the highest affinity Abs, recent new findings have made us realize that these GC responses also maintain a diverse population of antigen-specific B cells. Thus, affinity maturation does not necessarily involve radical loss of diversity (2). This point is particularly important from the viewpoint of development of broadly protective memory B cells during the GC reaction. Hence, we first discuss positive selection of high-affinity GC B cells, clonal diversity in the GC and memory B cell differentiation mechanisms, mainly by using model antigens.

### Positive Selection of High-Affinity GC B Cells

Before entering the GC, activated B cells compete for follicular helper T (Tfh) cell help at the T-B border based on the amount of peptide-MHC class II (MHC-II) presented by the B cells to the Tfh cells. Hence, the success of B cells competing for early Tfh help depends on their frequency and their B cell receptor (BCR) affinity for antigens (14–17). Rare B cells, such as broadly neutralizing antibody (bnAb) precursor B cells with low affinity (18), may be excluded from entering the GC at this early Tfh cell checkpoint.

After the B cells join the GC reaction, inter-clonal (between clones with different V(D)J rearrangements harboring variable epitope specificities and affinities) as well as intra-clonal (between SHM variants originating from the same clone) competition begins to take place. Previous model hapten studies showed that hapten-driven GCs tend to become more clonally homogeneous over time and reflect only the intra-clonal competition; variable-sized expansions of particular SHM variants of the one particular B cell clone (2).

**Figure 1:**
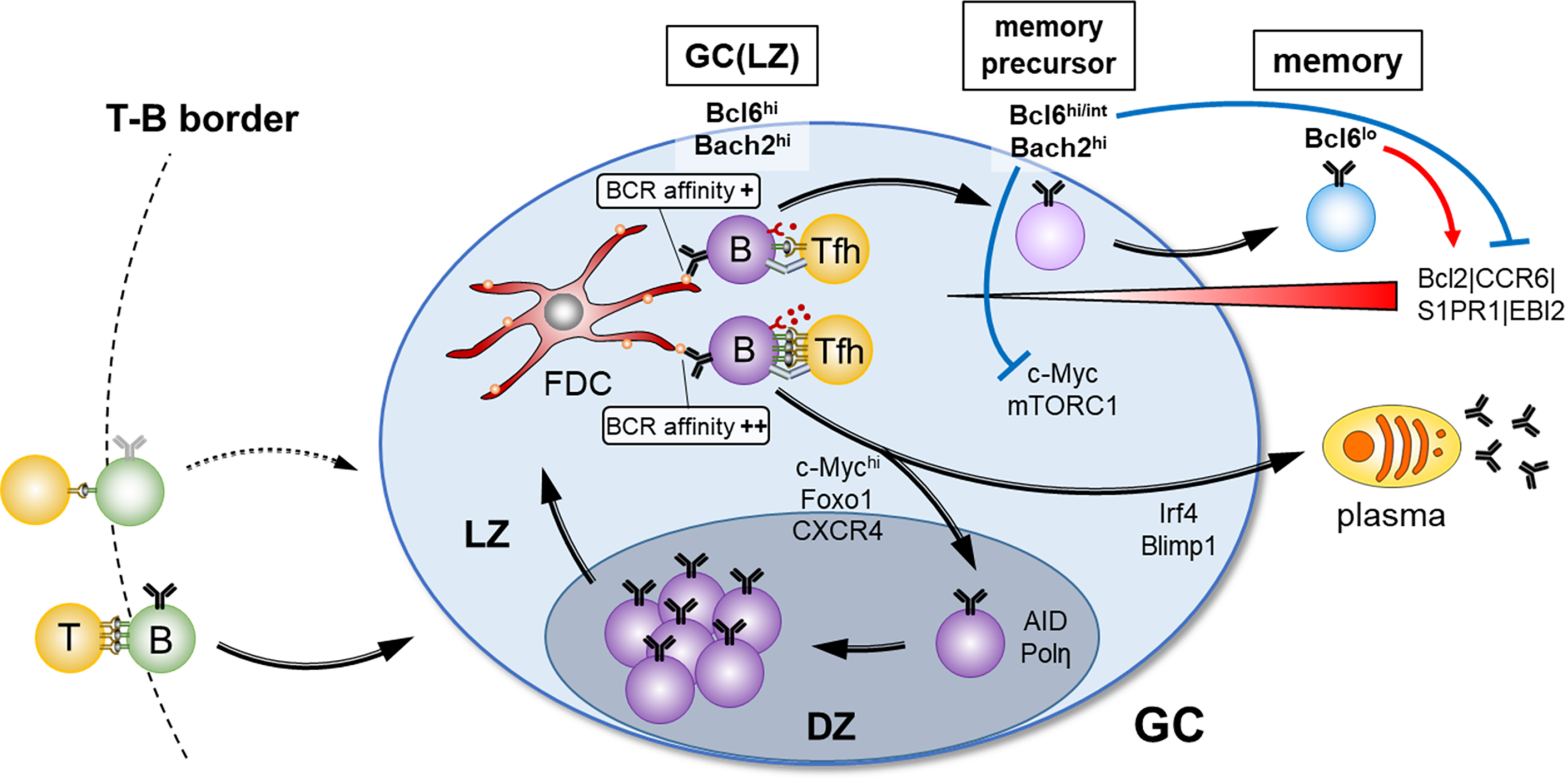
Overview of the GC selection and the factors for memory B cell fate. After antigen-activated B- and T cell contact at the T-B border in secondary lymphoid organs, B cells enter into GC reaction. Clonal expansion and BCR diversification occur in the DZ, and affinity selection for the fate decision of B cell differentiation through interaction with FDCs and Tfh cells takes place in the LZ. Strong T cell help due to high BCR affinity determines the plasma cell fate or the reentry to the DZ, whereas weak T cell help due to low BCR affinity favors memory B cell fate. Suppression of mTORC1 activity and c-Myc expression mediated by high Bach2 expression, and a provision of survival signals mediated by down-regulation of Bcl6 are the key drivers for GC B cells to adopt a memory B cell fate.

A fundamental characteristic of the GC reaction is that GC B cells constantly migrate between microanatomical compartments (**Figure 1**). The GC is classically defined as the dark zone (DZ) and light zone (LZ). SHM and subsequent cellular selection occur in the DZ and the LZ, respectively. The DZ consists primarily of highly proliferating B cells, expressing high levels of AID and error-prone DNA polymerase η, while the LZ is composed of GC B cells, follicular dendritic cells (FDCs), and Tfh cells. Intravital photoactivation experiments revealed that although the LZ is constantly being repopulated by massive immigration from the DZ, at a rate of 50% of DZ cells transitioning to the LZ over a period of 4 hrs, less than 10% of LZ cells return to the DZ over a period of 6 hrs (19). Overall, in contrast to the DZ to LZ transition, the LZ to DZ transition is a highly selective process; only about 10% of B cells that arrive to the LZ are selected to re-enter the DZ. A small population of the LZ exits the GC as memory B cells and plasma cells, and the majority of the remaining cells die by apoptosis. Thus, how these re-entering cells are positively selected is one of the key points for affinity maturation. B cells with damaged BCRs undergo apoptosis in the DZ and cells failing to receive sufficient helper signals in the LZ are also thought to undergo cell death (discussed in detail below). The overall rate of cell death is such that up to half of all GC B cells die every 6 hrs, suggesting that GC B cells possess specific systems for provision of high proliferation together with high apoptotic capabilities (20). Of note, a recent study defined an additional group of proliferating DZ cells enriched for G2/M phases of the cell cycle as gray zone GC B cells (21).

In regard to antigen-based signals involved in affinity maturation, the LZ GC B cells are well-equipped with two sensing systems; one is through the BCR and the other is delivered by cognate interaction with Tfh cells. The BCR recognizes antigen, which is displayed on FDCs as antigen-Ab complexes, through their binding to FcγRII and complement receptor 2 on FDCs. The BCR recognizes the antigen on FDCs and acts as a signal-transducing receptor as well as an endocytic receptor. Therefore, GC B cells utilize the BCR to retrieve antigen in an affinity-dependent manner, and present processed peptides/MHC-II complexes to Tfh cells, thus providing a mechanism by which Tfh cells can indirectly sense BCR affinity (2). Then, Tfh cells provide T cell help, mainly CD40 and IL-4/IL-21 cytokine signals, to cognate GC B cells. Indeed, recent studies suggested that progressive differentiation of Tfh cells regulate the GC response through IL-4 and IL-21 secretion, and Tfh-derived IL-4 plays a critical role in the expansion of rare broadly neutralizing GC B cell clone (22, 23). Thus, the importance of Tfh cell help in GC B cell selection is clear, but it is not the only factor. GC B cells with MHC-II haplo-insufficiency compete equivalently to wild type cells under conditions of physiological antigen concentration (16). Thus, both BCR signaling and Tfh cell help signals appear to be integrated in GC B cells to determine survival and proliferation (24). Different from naïve B cells, in the case of GC B cells, CD40L-CD40 engagement triggers NF-κB, and BCR antigen signaling engages PI3K signaling (25).

Antigen-loaded B cells in the LZ begin to achieve clonal dominance through accelerated cell division and increased biomass accumulation, which are c-Myc and mTORC1-dependent, respectively (26). For cell division, the strength of the Tfh cell signal delivered in the LZ is directly proportional to the levels of c-Myc and this then dictates the number of cell divisions that occur in the DZ, supporting the previously proposed “timer model” to explain how many times DZ GC B cell could proliferate (27). In addition, a recent study has demonstrated that cyclin D3 plays a specialized role in the GC cell cycle transition from G1- to S-phase (28).

### Clonal Diversity in the GC

As described above, previous studies using hapten-immunization showed that the Ab responses are strongly focused on the hapten and heavily dominated by stereotypical V genes (2, 29, 30). Apart from this occasion, in the case of viral infection and vaccination, many clones with distinct V(D)J rearrangements participate initially and competition occurs among B cells derived from various clones, too.

**Figure 2:**
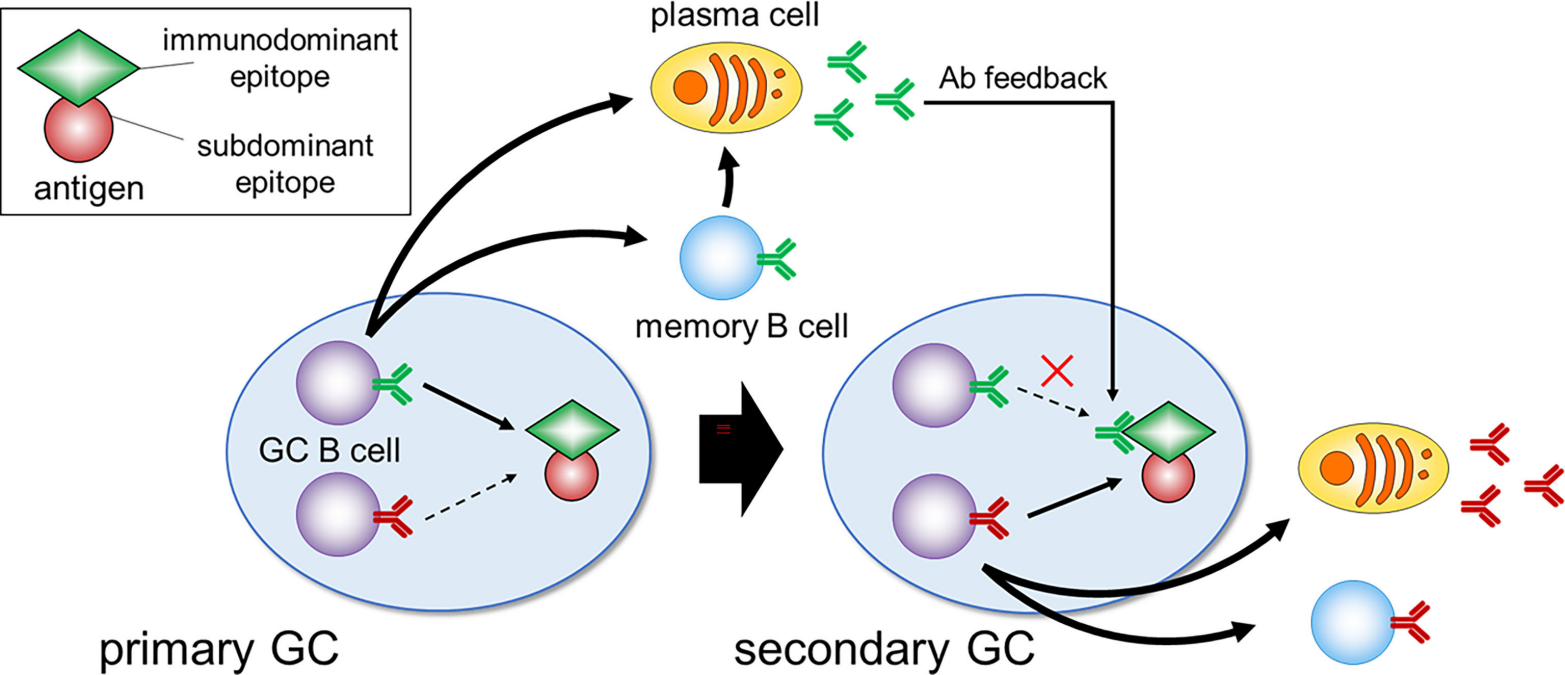
Immunodominance and Ab feedback. In the primary GC, B cell clones specific for the immunodominant epitopes dominate and give rise to plasma cells that secrete Ab. During the secondary GC response, this Ab masks its own epitope, which can suppress the expansion of immunodominant clones and enhance the selection of clones specific for less accessible subdominant epitopes.

Two studies aimed to circumvent this hapten-related issue by applying technologies that allow examination of the more diverse GC responses to un-haptenated protein antigens (31, 32). Although it was previously thought that only one or a few clones participate in one GC, as demonstrated by employing hapten conjugates, these new studies showed that tens-hundreds of clones participate in one GC at the early phase. Among several GCs formed after protein-immunization, rapid and massive expansion of specific higher-affinity SHM variants, as observed in the hapten model, leads to substantial loss of diversity in a subset of GCs, while other GCs in the same lymphoid tissue continue the affinity maturation process and still retain substantial clonal diversity. Since individual GCs are spatially separated, this model explains how GCs support a diversity of antigen-specific clones against complex protein antigens (20). Other mechanisms can be also envisaged. For instance, clonal diversity may be promoted by antibody-mediated feedback (33–35), as dominant GC clones with BCRs specific for a particular antigen epitope give rise to plasma cells that secrete Ab. This Ab then masks its own epitope, thereafter halting proliferation of the already expanded clone, while enhancing the selection of other clones that bind to different epitopes (**Figure 2**). Further investigation of the mechanisms allowing sustained diversity in GCs is important as they have wide implications, for example in the context of efforts to generate bnAb responses by iterative immunization.

### Differentiation of GC B Cells Into Memory B Cells

An early study in which transgenic overexpression of the anti-apoptotic factor Bcl2 resulted in a marked increase of low affinity B cells in both GC and memory compartments without impairing the selection of high affinity plasma cells (36), suggested that the differentiation of GC B cells to memory B cells is a default process. However, this notion has been challenged by recent studies employing mono-epitope hapten or HEL antigens, which provided evidence for the existence of instructive regulations in memory B cell selection from the GC compartment (4, 6). First, contemporaneous comparison of memory and GC B cells indicates that the memory B cell pool arises predominantly from the low affinity cells. Second, consistent with another study (5), the memory B cell pool is generated early during an immune response. Considering that affinity maturation in the GC is still continuing after stopping memory cell generation, this would also contribute to the observed accumulation of overall less SHM and the preponderance of low affinity B cells in the memory B cell compartment in the case of mono-epitope antigen. A recent study employing poly-epitope protein antigens has also reinforced these conclusions, except that in this case the memory B cells are generated throughout the immune response (7). A probable explanation for this difference is that, unlike mono-epitope systems, in the case of a poly-epitope system, some clones keep expanding in the late GC reaction, thereby also generating memory B cells at later time point. Furthermore, this study showed that, like in the case of flavivirus-specific memory B cell generation (37), B cells with low affinity germline BCRs are prone to be selected into memory B cells. Given that the germline BCR of bnAbs usually has very low affinity for the native antigens of influenza virus, this may be one of the reasons why broadly reactive clones against influenza virus can exist in the memory fraction (38).

The question then arises of what is the molecular nature of the above instruction program for memory B cell generation. In contrast to plasma cell precursors, memory B cell precursors within the GC are no longer cycling (6, 39, 40). In addition, these memory precursors reside at the edge of the LZ (41). Thus, at least, three inter-connected processes seem to be required for the transition from GC to memory B cells; i) stopping proliferation in the DZ; ii) returning to the LZ and being in the process of exiting the GC; iii) entering the quiescent (G0) stage with acquisition of survival signals.

As mentioned above, once positively selected GC B cells possessing high levels of c-Myc expression and mTORC1 activity reenter the DZ, they start proliferation, accompanied by a stepwise decline of the level of c-Myc and active mTORC1, depending upon their proliferation numbers. Trafficking back to the LZ requires decay of both mTORC1 activity and c-Myc expression. Since rapamycin treatment downregulates Foxo1 expression (26), decay of mTORC1 activity is likely to downregulate CXCR4, thereby promoting the return from the DZ to the LZ. After their return to the LZ, memory B cell precursors should maintain high Bach2 expression, because of receiving low T cell help, and dampen mTORC1 activity and c-Myc expression, which is thought to be one of the mandatory steps for transition to memory B cells (**Figure 1**). In support of this, Bach2-deficient GC B cells manifested constitutively active mTORC1 and c-Myc expression, thereby resulting in hyper-proliferation, and subsequent inability to generate memory B cells (40). Conversely, impairment of the interaction of c-Myc and MIZ1 skewed the system towards memory B cell generation (42). c-Myc and MIZ1 form a transcriptional repressor complex for MIZ1 target genes, such as cell cycle inhibitors. The absence of the c-Myc-MIZ1 interaction releases this repression, resulting in impaired cell cycle entry of positively selected GC B cells, supporting the notion that the inhibition of the c-Myc activity contributes to memory B cell generation.

The above anti-proliferative activity is required but appears not to be sufficient for development of memory B cells. Since low-affinity B cells receive low T cell help, it has been previously thought that these GC B cells undergo apoptosis. Therefore, the question arose of how such memory precursor cells with low affinity are prevented from dying and are able to differentiate into mature memory B cells. A recent detailed GC B cell analysis of Bcl2 transgenic mice provided us a hint to answer this question. In these mice, aberrant populations of seemingly quiescent cells arise that express markers of memory precursor cells (CD38^+^ and CCR6^+^) (43). Hence, we speculated that, in physiological settings, initiation of Bcl2 up-regulation might take place in these precursor cells, thereby giving them a survival advantage. This idea was directly tested, demonstrating that it is indeed the case (40). Then, in regard to how to initiate Bcl2 expression, we considered that differentiation of GC B cells to memory B cells to a large extent involves reversion to the gene expression profile they possessed prior to differentiation into GC B cells. This includes re-expression of genes, *Ccr6*, *Gpr183* (EBI2), *S1pr1*, and *Bcl2*, each of which is known to be directly repressed by Bcl6 (44–46). Given that *Il21* knockout mice showed down-regulated Bcl6 in GC B cells (47), it is likely that memory precursor cells limit access to IL-21, which, in turn, begins the process of down-regulation of Bcl6. Consequently, Bcl2 is up-regulated, thereby providing survival signals. This Bcl6 down-regulation is further augmented by a recently identified transcription factor Hhex during the maturation processes in GC-mediated memory B cells (48). Hence, we would propose the existence of two key drivers for differentiation of GC B cells to memory cells; one is high expression of Bach2, antagonizing the c-Myc and mTORC1 pathways, and the second is down-regulation of Bcl6, releasing its repression of Bcl2 and providing survival signals. Importantly, this model seems to well explain why memory precursor cells, despite receiving low T cell help, acquire the survival signal (**Figure 1**).

## Generation of Broadly-Neutralizing Memory B Cells and Their Recall

Recent discoveries of bnAbs for HIV and influenza virus have provided new outlooks in the vaccine field and highlighted the need to understand how such bnAb precursors enter the GCs, thereafter, creating memory B cells expressing high quality bnAbs (18).

Abs against the influenza virus surface glycoprotein hemagglutinin (HA) are a key correlate of protection (49). HA is composed of head- and stem-regions; in contrast to the structural changes in the head-region by antigenic drift and shift, the stem-region is well conserved, thereby making it a good target for generating cross-reactive bnAbs. However, in normal immune settings, most Abs are generated against the HA head-region, because of its immune dominance, while the stem-region acts as an immune subdominant epitope. Three potential mechanisms are thought to explain the subdominance of the stem Ab response (18, 50, 51). First, most of the germline BCRs of the stem Abs have very low affinity for the native antigen. Second, overlapping with the first possibility, these Abs have limited access to the stem epitope due to a steric hindrance. Finally, many of the stem Abs are polyreactive towards dsDNA, LPS, and insulin, potentially having inherent self-reactivity (52).

Nevertheless, it was observed that, upon vaccination with a novel influenza virus, the 2009 pandemic H1N1, some, but not all individuals generated broadly reactive HA stem-binding Abs (10, 53). Considering that almost all the induced anti-stem plasmablasts were mutated, these data indicate that, prior to vaccination, anti-stem memory B cells existed in individuals already exposed to previous infection of other types of influenza viruses, and that these memory B cells got activated after novel pandemic H1N1 vaccination. The conclusions from human vaccination data are further strengthened by a mouse infection model (9). Infection of naïve mice with H1N1 Narita strain influenza virus generated anti-stem memory B cells, but very low levels of anti-stem Abs, reflecting the long-lived plasma cell compartment, indicating a relative enrichment of anti-stem clones in the memory B cell rather than long-lived plasma cell compartment. Then, upon secondary infection with a drifted virus (H1N1 PR8 strain), such anti-stem memory B cells were promptly activated and differentiated to plasmablasts, thereby contributing to protection against PR8 virus infection. Furthermore, single cell Ab analysis showed that these anti-stem memory B cells are generated through GC reactions during primary Narita virus infection, thereby manifesting affinity-maturation and breadth at least to some extent. Thus, it is important to understand how GC-experienced (mature mutated) anti-stem memory B cells can be generated in naïve and recall conditions.

### Generation of Anti-Stem Memory B Cells Under Naïve Conditions

In the naïve state, the key to generating mature influenza anti-stem bnAb memory B cells depend upon several aspects; recruitment of appropriate naïve precursors into the GC; positive selection of appropriate clones during the GC reaction; exiting from the GC as long-lived memory B cells. Considering that, during the GC processes, selection into memory B cells is less stringent than that for long-lived plasma cells, major hurdles seem to be the GC recruitment of rare anti-stem bnAb precursors and the duration of rare bnAb B cells in the GC.

In regard to the recruitment to the GCs, difficulties are due to; i) a very low affinity of the naïve anti-stem precursors for the native HA antigen; ii) the subsequent problem in receiving sufficient T cell help; iii) the possibly anergic state of these precursor B cells because of poly-reactivity (54). In regard to problem iii), studies using the model antigen HEL system provided significant insight into how we can awaken such anergic B cells (55, 56). The key to activating anergic B cells is applying particulate type immunogens decorated with high densities of a closely related foreign and higher affinity antigen. In regard to point i), in the HIV case, it was shown that the quantity and affinity of the precursor naïve B cells are important for their entry into the GC. In addition, multimerization of antigen increased GC recruitment of rare naïve precursors by 200- to 500-fold compared to the equivalent monomeric antigen (15). Hence, like the immunogen in the HIV case, designing immunogen variants with higher affinity for rare anti-stem precursor Ab and their multimerization is one way to overcome points i) and iii).

How can we tackle point ii)? Indeed, several HIV human cohort data indicated the positive association between Tfh cells and anti-HIV bnAbs; frequencies of PD-1^+^CXCR3^-^CXCR5^+^, or PD-1^lo^CXCR3^+^CXCR5^+^ CD4 T cells correlated with HIV neutralization breadth (57, 58). This raised the question of whether this is a simple correlation or a causal association. This issue has been approached in the mouse by employing BCR (VRC01 germline version) knock-in B cells and transgenic TCR T cells (59). Since, in the case of influenza and HIV bnAb responses, immunodominance is one of the key issues, this study was particularly designed to address whether increasing accessibility to T cell help preferentially enhances the rare immune-subdominant bnAb precursor B cell responses. GC occupancy by rare bnAb precursor VRC01 B cells was improved by increasing the quantity of HIV Env-specific CD4 T cells, even in the presence of the endogenous immunodominant B cell precursors. Moreover, the action point of this T cell help seems to be on the recruitment of VRC01 B cells into GCs. Because this study utilized modified high affinity antigen for bnAb precursor B cells (*K*_D_ value of ~ 0.1 μM), it was concluded that, as long as high affinity antigen for bnAb precursor B cells is used, a high quantity of T cell help can promote recruitment of these rare B cells into GCs. However, it remains to be addressed what increasing T cell help does in conditions of weaker affinity antigen for bnAb precursors. Insufficient T cell help is also likely to occur in the case of influenza anti-stem bnAb precursor B cell responses. In fact, in mice, when employing only the stem region as an antigen, recruitment of polyclonal anti-stem B cells into the GC was very rare, whereas conjugation of this antigen to KLH, which contains strong T cell epitopes, resulted in better recruitment into the GC (60). Thus, conjugation of appropriate T cell epitopes to a B cell antigen is worth considering. In addition to antigen, development of good adjuvants for inducing Tfh cells is also important. Indeed, lipid nanoparticles used for mRNA vaccines have recently been shown to facilitate Tfh cell generation, presumably through enhancing IL-6 production (37).

Given that it takes years for bnAbs to emerge during infection (61), generation of rare high quality bnAb-producing clones may require prolonged GC responses, which may simply reflect the need for many rounds of SHM (3). Alternatively, it is also possible that prolonged GCs include more clonally permissive B cells over time. As discussed above, limited Tfh cell help or epitope-masking by generated Abs may be redirected towards B cell clones that bind non-dominant epitopes. In addition, changing Tfh cell clones during GC responses also may contribute to such redirection towards different B cell clones. For maintaining GC responses, as proposed in the HIV vaccination system, slow continuous delivery of native antigen might be one approach (62). This delivery method directed the response away from non-neutralizing Abs, which were dominantly present on degraded HIV Env trimers, instead towards a neutralizing Ab response. This method, in addition to continuous provision of antigen, provides more of it in native conformational form, therefore causing the observed biological effects. In the case of influenza vaccination, recently developed oil-in-water adjuvant (AS03) might utilize similar mechanisms, thereby increasing cross-reactive anti-influenza Ab responses (52, 63). This oil-in-water adjuvant functions to emulsify viral antigens within the adjuvant, which may protect them from degradation and may allow for the delivery of native antigens to lymph nodes. Collectively, delivery of antigen in its native form seems to be one of the important factors for maintaining GC reactions.

### Behavior of Anti-Stem B Cells During Recall Responses

Between yearly vaccination and seasonal infection, individuals repeatedly mount an immune response against the influenza virus. Hence, it is important to understand how such immune history, formed by previous infection/vaccination, affects the *de novo* immune response induced by the current vaccine. Mouse studies traced the fate of HA-induced memory B cells after repeated immunization with the same antigen and demonstrated that more than 90% of B cells in the secondary GCs have no prior GC experience; many of them are likely derived from naïve B cells (64). Thus, memory B cell reentry into GCs is rare upon repeated vaccination with the same antigen.

Then, the question became, what is the outcome when a variant of the original antigen was used for the second immunization, a situation similar to what occurs with annual influenza vaccination. By using fine-needle aspiration for obtaining human immune cells from lymph nodes, GCs were analyzed from people immunized with the 2018-2019 influenza seasonal vaccine (65). This analysis revealed that some of the GC B cell repertoire was shared with that of the *de novo* generated plasmablasts. This suggests that memory B cells, formed in response to a different earlier influenza strain, proliferated in response to the 2018-2019 vaccine, probably corresponding to a new influenza strain to these individuals, and that the progeny cells became plasmablasts or entered the GCs. In contrast, the GC B cells that did not share the Ab repertoire with plasmablasts were likely derived from naïve B cells. Importantly, the BCR repertoire from the memory-derived GC cells was directed toward cross-reactive epitopes, whereas those from naïve-derived GC cells were strain-specific. Although it remains to be determined whether cross-reactive and/or strain-specific GC B cells joined the long-lived memory B cell compartment, these human data suggest the encouraging possibility that once memory B cells with bnAb are generated, they can be recruited to secondary GCs. These 2018-2019 vaccination data appear to be consistent with the aforementioned 2009 pandemic H1N1 vaccination cohort data indicating that pre-existing anti-stem memory B cells are activated by vaccination with a novel type of influenza virus (10). However, a previous longitudinal study after vaccination with a current influenza revealed no overall increase in SHM in memory B cells over time (66), suggesting either that, despite entering the GCs, the entry efficiency is low, or that the step from GC to memory B cells is limiting.

In regard to entering the GC, one key difference between mouse and human data described above is vaccination with the same or variant antigens, suggesting that the extent the second antigen differs from the first is one of the key factors that dictates which B cells are recalled by influenza vaccination and recruited into the secondary GCs. Below we will discuss the potential mechanisms.

It was previously thought that memory B cells have higher affinity and are present at higher frequencies than naïve B cells specific for the same antigen. However, given the current view that memory B cells possess a more diverse range of affinities, it is possible that the numbers of memory B cells with higher affinities than naïve B cells might be smaller than expected. In addition, several functionally distinct memory B cell subsets are generated, among which the CD80^+^CD273^+^ subset is more prone to differentiate into plasmablasts rather than enter the GC (67–69). Thus, the actual numbers of memory B cells that are competent for entering the GC might be small. Such low numbers of competent memory B cells might be one of the reasons of why recruitment of memory B cells into secondary GCs is unexpectedly low. In this case, as discussed in section 2-1, affinity and multiplicity of the B cell antigen and T cell epitopes should be carefully considered.

One of the big differences in the immune state between naïve and memory cells is that due to already establishing long-lived plasma cells upon primary vaccination/infection, high titer and high affinity Abs for the primary antigen preexist prior to the secondary vaccination. Among many effector functions of Abs, the following three activities presumably affect subsequent recall humoral responses; 1) rapid antigen clearance, e.g., *via* macrophage Fc and complement receptors; 2) immune complex formation (70) and subsequent antigen presentation on FDCs; 3) epitope masking (71). Although the relative involvement and importance of these three mechanisms in recall responses have not been carefully addressed, epitope masking is likely to occur, evidenced by recent data employing malaria vaccination (72). Abs against *Plasmodium falciparum* circumsporozoite protein (PfCSP) plateaued after two immunizations with the same antigen and these Abs masked the epitope, thereby limiting immunodominant B cell responses upon the third immunization with the same antigen. They allowed subdominant responses toward distinct epitopes, thereby contributing to broadening the spectrum of vaccine-induced Abs (**Figure 2**).

**Figure 3:**
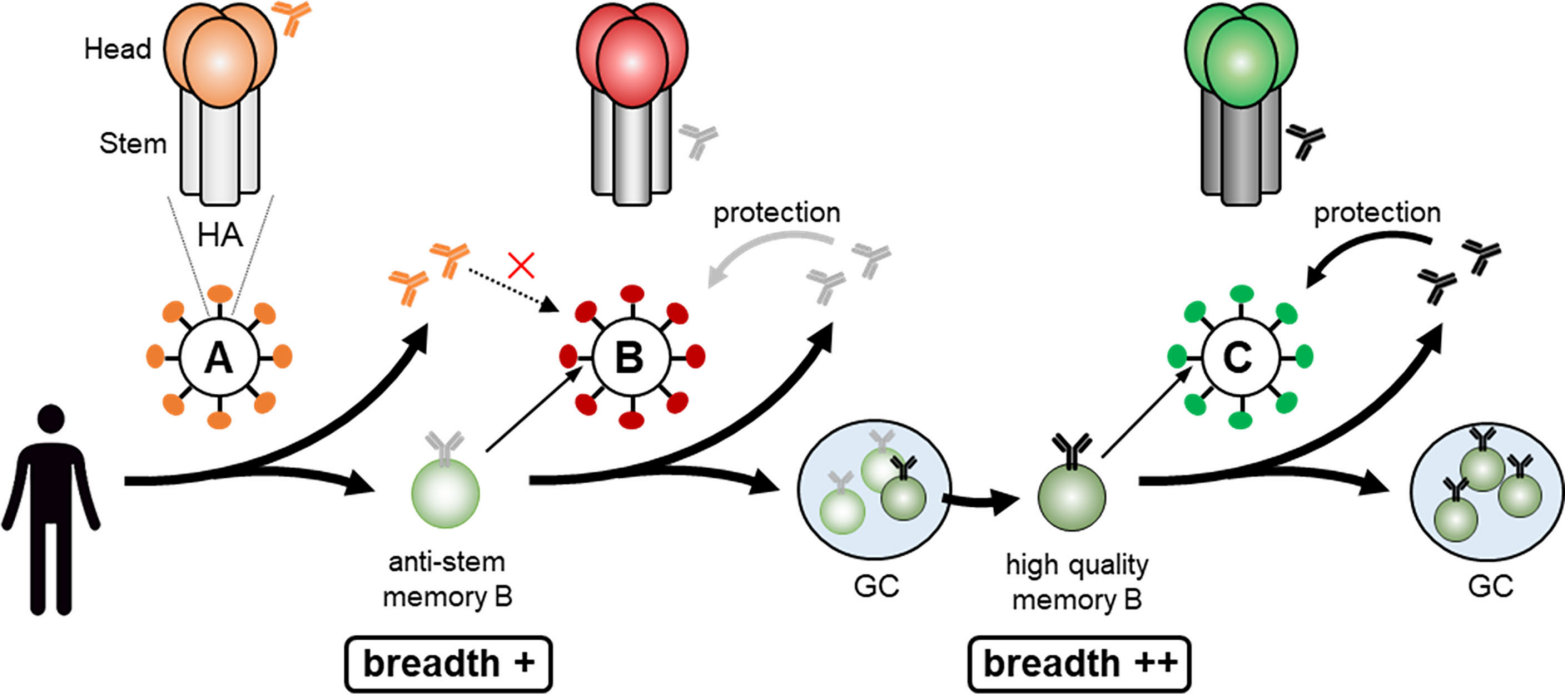
Making high quality anti-stem memory B cell. Infection with the virus **(A)** results in generation of anti-stem memory B cells and low levels of anti-stem Abs. In this case, as there are no stem epitope masking Abs, anti-stem memory B cells are activated upon virus **(B)** infection, quickly produce anti-stem Abs, along with acquiring further breadth by entering GCs and subsequently generating more mature high quality memory B cells. Once the further distant virus **(C)** infects, high quality anti-stem memory B cells would play a significant role in protection from the virus **(C)**.

Assuming that, to simplify the model, influenza HA has five dominant epitopes in the head, which change in variant viruses, and one conserved subdominant epitope in the stem (73), based upon the epitope masking mechanism, the following scenario can be envisaged (**Figure 3**). If the antigenic distance between the virus (A) that the individual was initially exposed to and the current exposure virus (B) is large, pre-existing anti-head Abs against virus (A) cannot protect from the (B) virus. After infection/vaccination with the virus (A), anti-stem memory B cells are probably also generated but, as seen in the mouse infection case (9), levels of anti-stem Abs are very low. In this case, upon virus (B) infection, because there are no epitope masking Abs for the stem epitope, anti-stem memory B cells are activated. Simultaneously, GC and memory B cells directed towards the unique head region of the new (B) virus are generated from naïve B cells. The anti-stem memory B cells swiftly produce plasmablasts, along with entering GCs and subsequently generating more mature memory B cells. Thus, this individual can be protected from (B) virus due to the *de novo* promptly generated anti-stem Abs and will be prepared for the subsequent variant virus (C) infection due to generating more mature mutated anti-stem memory B cells by acquiring further breadth in GCs. Once the further distant virus (C) infects, even though the anti-stem Abs have declined, high quality anti-stem memory B cells would play a significant role in protection from the (C) virus. Validating this scenario needs further study but, to make high quality anti-stem memory B cells, limiting epitope masking by anti-stem Abs seems to be another option.

## Concluding Remark

Considerable advances have been made in elucidating the cellular basis and key drivers regulating GC-derived memory B cell differentiation and activation in recent years (74, 75), which can explain the mechanism how the memory B cell pool can deal with the variant viruses. Generation of high quality broadly protective memory B cells against conserved viral epitopes remains a continuous challenge to provide long-lasting and cross-protective immunological memory, but the recent progress in this field will facilitate the development of better Ab-based universal vaccine design strategies.

## Author Contributions

TI, RS, and TK contributed to conception of the study. TI and TK wrote the manuscript. All authors contributed to the article and approved the submitted version.

## Funding

This work was supported by grants from JSPS KAKENHI (JP21H02749), Takeda Science Foundation, the Naito Foundation, and the Sumitomo Foundation to TI, grants from JSPS KAKENHI (JP21H02740) to RS, and grants from JSPS KAKENHI (JP19H01028) to TK.

## Conflict of Interest

The authors declare that the research was conducted in the absence of any commercial or financial relationships that could be construed as a potential conflict of interest.

## Publisher’s Note

All claims expressed in this article are solely those of the authors and do not necessarily represent those of their affiliated organizations, or those of the publisher, the editors and the reviewers. Any product that may be evaluated in this article, or claim that may be made by its manufacturer, is not guaranteed or endorsed by the publisher.
